# Tongue as a first-line immune organ?

**DOI:** 10.1007/s13238-020-00802-x

**Published:** 2021-01-09

**Authors:** Jane Y. Wu

**Affiliations:** Department of Neurology, Center for Genetic Medicine, Lurie Cancer Center, Northwestern University Feinberg School of Medicine, Chicago, USA

The tongue is an organ strategically situated at the beginning of the gastrointestinal (GI) system, yet it has been remarkably understudied. Not only there is no separate subspecialty dedicated to the tongue, it is even excluded from 27 human organs/tissues thoroughly archived in the NCBI gene expression database. Almost none of my physician colleagues in Western medicine have paid attention to it, except a few who study tongue cancer. The tongue is typically described as a muscular organ important for taste, mastication, speech and sensation. Other than its development, anatomy/gross structural analyses and taste function (for recent reviews, see Roper and Chaudhari, 2017), the human tongue is poorly studied in Western medicine, in particular, in terms of its roles in systemic diseases. In traditional Chinese medicine (TCM), however, the tongue holds a special place. Assessing the “tongue coating”, “tongue body” and morphological features is one of the most critical skills that TCM doctors have relied on for disease diagnoses for thousands of years before the advent of Western Medicine.

It is well known that vertebrate tongues are rich in nerve innervation, blood supply and lymphatic drainage. Nonetheless, the human tongue as a sensory organ, is much less studied in comparison with other sensory organs such as eyes, ears or nose. The role of the tongue in systemic diseases or immunity has been underestimated, if not entirely overlooked. Here, I propose that the tongue serves as a first-line immune organ and plays a critical role in defense against various diseases. This notion is supported by accumulating evidence, although data still remain sporadic and largely anecdotal. In the recent pandemic COVID-19 outbreaks around the world, there have been prevalent reports of losses of taste and smell in patients infected with SARScoV2, bringing the potential involvement of the tongue in viral infection into our focus again.

Several lines of evidence support my hypothesis that the human tongue acts as an immune organ. First, the tongue epithelium acts as an efficient defense barrier against vast amounts of allergens, immunogens and pathogens. It remains unclear how the lining of the tongue epithelium functions in such defense, in particular, in comparison with other epithelia, for example, intestinal or respiratory epithelium. Second, immune cells are located in the tongue epithelium in normal tongue tissues from healthy human subjects (Gondak et al., 2012). Similarly, various types of immune cells, ranging from antigen presenting cells, macrophages, dendritic cells, mast cells and lymphocytes have been detected in tongue epithelia of other species (Shaikh et al., 1997; Thirion-Delalande et al., 2017). It has been reported that Langerhans cells were frequently detected in the tongue epithelial samples of control healthy subjects. Interestingly, these tongue-resident dendritic cells were depleted in patients affected by Acquired Immune Deficiency Syndrome (AIDS) (Gondak et al., 2012). It should be noted that a complete catalogue of different cell types present in the human tongue is still missing and that the composition of the resident immune cell repertoire in the human tongue remains to be defined. Third, immune cells in the tongue epithelia can be efficiently activated, similar to those in the intestine (Shaikh et al., 1997; Thirion-Delalande et al., 2017). Intriguingly, taste receptors have been reported to mediate immune responses in respiratory epithelia (Freund et al., 2018; Carey and Lee, 2019; references within), raising the possibility that such taste receptors also modulate immune responses in the tongue epithelia and in other organs or tissues. On the other hand, receptors critical for innate immunity may function in mediating taste responses. For example, Toll-like receptor 4 has been shown to mediate fat, sugar, and umami taste preference (Camandola and Mattson, 2017). In addition, taste receptors have been detected in human peripheral leukocytes (Orsmark-Pietras et al., 2013; Malki et al., 2015) and lung macrophages (Grassin-Delyle et al., 2019). Fourth, taste bud cells and leukocytes both express a subset of taste receptors (Roper and Chaudhari, 2017), supporting the idea that taste receptor ligands may play a role in mediating communication between the tongue and other classical immune organs.

Finally, sublingual vaccination in animals, including topical application, has been demonstrated to elicit not only mucosal response but also systemic immune responses, including humoral and effector T cell responses in both rodents and primates (Cuburu et al., 2007; Czerkinsky et al., 2011; Jones et al., 2019). Sublingual injection of a DNA-based vaccine was shown to be as effective as intramuscular or intradermal injection in mice (McCluskie et al., 1999). Recent studies have demonstrated the safety of sublingual immunotherapy among AIDS patients (Iemoli et al., 2016). In light of the observation that overreactive and excessive immune and inflammatory responses may contribute to the lethality of certain viruses, including SARScoV2 (Iemoli et al., 2016; Cao, 2020; Park and Iwasaki, 2020), immune modulatory nucleic acid-based vaccines, such as those being developed for allergies (reviewed in Scheiblhofer et al., 2018), may help in combating such lethal viruses. In contrast to nasal administration, sublingual delivery may have additional advantages of limiting or avoiding redirection of antigens and/or adjuvants to the brain. Further studies are necessary to determine which route, uptake by blood vessels or by lingual mucosa, plays a major role in sublingual immunization. Nonetheless, emerging data suggest sublingual delivery as a potential route for vaccine delivery as well as immunotherapies and warrant further systematic and rigorous studies in humans. It is conceivable that resident immune cells in the tongue, both innate type and adaptive type, may communicate with other classic immune organs, such as those inside or outside of the GI tract, by both conventional and unidentified mechanisms.

Communication between the nervous system and immune system has been a field of active research. The potential crosstalk between taste and immune signaling pathways remains an area largely unexplored. A number of questions remain to be answered. For example, whether taste signaling regulates immune responses by direct or indirect mechanisms; or whether immune signaling impacts on taste sensation.

In summary, I propose the hypothesis that the human tongue may act as a first-line immune organ and call for systematic and rigorous investigation to test this hypothesis using interdisciplinary approaches. Such studies will not only advance our understanding of fundamental mechanisms underlying human immunity but also likely enhance our diagnostic and therapeutic approaches to a wide range of diseases.
